# A corpus of potentially contradictory research claims from cardiovascular research abstracts

**DOI:** 10.1186/s13326-016-0083-z

**Published:** 2016-06-07

**Authors:** Abdulaziz Alamri, Mark Stevenson

**Affiliations:** Department of Computer Science, The University of Sheffield, Sheffield, UK

**Keywords:** Contradictory claims, Natural language processing

## Abstract

**Background:**

Research literature in biomedicine and related fields contains a huge number of claims, such as the effectiveness of treatments. These claims are not always consistent and may even contradict each other. Being able to identify contradictory claims is important for those who rely on the biomedical literature. Automated methods to identify and resolve them are required to cope with the amount of information available. However, research in this area has been hampered by a lack of suitable resources. We describe a methodology to develop a corpus which addresses this gap by providing examples of potentially contradictory claims and demonstrate how it can be applied to identify these claims from Medline abstracts related to the topic of cardiovascular disease.

**Methods:**

A set of systematic reviews concerned with four topics in cardiovascular disease were identified from Medline and analysed to determine whether the abstracts they reviewed contained contradictory research claims. For each review, annotators were asked to analyse these abstracts to identify claims within them that answered the question addressed in the review. The annotators were also asked to indicate how the claim related to that question and the type of the claim.

**Results:**

A total of 259 abstracts associated with 24 systematic reviews were used to form the corpus. Agreement between the annotators was high, suggesting that the information they provided is reliable.

**Conclusions:**

The paper describes a methodology for constructing a corpus containing contradictory research claims from the biomedical literature. The corpus is made available to enable further research into this area and support the development of automated approaches to contradiction identification.

## Background

The research literature in medicine is vast and increasing rapidly. These papers contain a massive amount of information, including claims about the research question being addressed. However, papers may not come to the same conclusion about a particular research question and claims in different papers may even contradict one another. Contradictory claims make it difficult to understand the current state of knowledge about a research question. Systematic reviews aim to avoid this problem by evaluating and assessing the evidence related to a particular research question, including contradictory claims, and presenting it in a summarised format. However, these are not available for all research questions and are also limited by the evidence that was available when the review was written.

Tools that support the automatic identification of contradictory claims would be useful for those that rely on biomedical literature. They could be used to highlight research claims that are contradicted by other research findings, assist in the creation of systematic reviews [1] and literature surveillance systems [2]. They would also be useful for automatic text mining applications which generally accept claims made within research literature as prima face correct. Despite this there has been little exploration into this problem. The work that has been carried out [3] focused on descriptions of molecular events in a corpus mainly generated from the events in the BioNLP09 corpus [4], and was restricted to a single indicator of contradiction, the use of negation. Outside the biomedical domain there have only been a few attempts to study the problem of contradiction identification independently of the more general problem of textual inference [5–7].

One of the reasons for this limited progress is a lack of suitable resources that can be used to develop and test approaches. Developing these resources is not straightforward given the volume of research that has been published and the difficulty of identifying contradictory claims within them. This paper presents an approach to developing a corpus containing examples of potentially contradictory research claims which are identified by making use of information found in systematic reviews. The corpus is designed to include a wider range of topics from the biomedical literature and wider range of linguistic phenomena that can be used to indicate contradiction (e.g. negation, use of antonyms and adjective polarity) than previous work.

Reviewing the published biomedical literature to identify the best available information related to biomedical questions is standard practice [2]. However, the literature may contain findings that contradict one another and investigators have shown a tendency to reproduce the findings of original research with contradictory claims. Consequently, editors and publishers attracted by these results tend to publish them faster than those with less significant findings, dubbed the *Proteus phenomenon* [8]. A good example of contradictions between research claims that have appeared in the biomedical research literature concerns the relation between aspirin and heart attack prevention.

Aspirin has been widely used as a pain killer and an effective drug for preventing blood clots. A conflict on its benefits started when doctors began prescribing a daily dosage to protect heart attack victims from further attacks. At that time there was no biomedical research to prove that this was effective. An attempt to investigate [9] found that aspirin was significantly beneficial in preventing heart attacks. However, a subsequent trial was less confident about that because it found little difference between the fatality rate of people who never used, seldom used or often used aspirin [10]. Another study [11] was compatible with that result as it failed to show the preventative role of aspirin on heart attacks. The first team, who found a significant benefit of aspirin on the heart, conducted another experiment [12] and reported a positive results that supported their first claim. The contradictions between aspirin research claims lasted 20 years, until researchers finally concluded that aspirin reduces the risk of non-fatal heart attacks, but its effects on other problems such as stroke are still unclear [13].

Other research topics such as the effectiveness of mammographies for discovering breast cancer or whether the Dalkon Shield caused pelvic infections are also rich with contradictory claims [13].

## Methods

This section discusses some of the key concepts used in this work, beginning with the definition of contradiction and followed by the types of claim.

### Defining contradiction

Contradiction has been defined as the existence of two or more incompatible propositions that describe the same fact [14]. In another words, two fragments of text, *T*_1_ and *T*_2_, are contradictory when they assert information about the same fact that cannot both be true at the same time. The problem of contradiction has previously been explored within work on textual entailment [5, 7, 15] where a common approach is to consider *T*_1_ and *T*_2_ to be contradictory when one of them entails the negation of the other. De Marneffe et al. [6] used a looser definition intended to be less restrictive: two fragments of text are contradictory when they are extremely unlikely to be true at the same time.

There has been some previous exploration of the problem of identifying contradictions in biomedical documents [3]. Contradiction was defined as two texts that describe events sharing certain attributes (e.g. theme, cause and anatomical location) but with different polarity. That work was restricted to statements about a very specific type of information (chemical interactions) and one way of expressing contradiction (negation).

This work also focusses on biomedical documents but uses a less restrictive definition of contradiction. Two texts, *T*_1_ and *T*_2_, are said to contradict when, for a given fact *F*, information inferred about *F* from *T*_1_ is unlikely to be true at the same time as information about *F* inferred from *T*_2_.

This definition of contradiction is based on inferences from statements being *unlikely to be true at the same time* rather than being *logically inconsistent*. This approach avoids the definition of contradiction being overly restrictive and has been used by previous work [6]. Research findings in scientific documents are often expressed cautiously, e.g. using hedges [16], reducing the chances of statements being logically inconsistent with one another. Nevertheless, researchers are often interested in obtaining as much information as possible about a research question of interest and are likely to be interested in statements which are unlikely to be simultaneously true.

The language used in biomedical documents tends to involve complex sentence structures with multiple facts described within the same sentence and it is therefore important to consider contradiction relative to a particular research question or fact. For example, sentences (2) and (4) in Table 1 would be considered contradictory in relation to some facts but not others. Sentence (2) states that fish intake does not prevent heart failure without providing information about the types of fish or population groups the assertion applies to. Sentence (4) asserts that fish intake does prevent heart failure for a particular population group (“older adults”) and types of fish (“tuna, broiled or baked”). The sentences would not be considered contradictory relative to the fact “consumption of fried fish prevents heart failure”, since both suggest that it does not. However, they would be considered contradictory if the fact being considered was “eating tuna prevent heart attack in older adults” since sentence (4) suggests that it does while sentence (2) suggests that it does not.

**Table 1:** Claims extracted from the abstracts described in Table 3

	Claim	PMID	Value	Type
1	In this large, population-based sample of African-American and white adults, whole-grain intake was associated with lower HF risk, whereas intake of eggs and high-fat dairy were associated with greater HF risk after adjustment for several confounders	18954578	YS	CAUS
2	Our findings do not support a major role for fish intake in the prevention of heart failure	19789394	NO	CAUS
3	Moderate consumption of fatty fish (1-2 servings per week) and marine omega-3 fatty acids were associated with a lower rate of first HF hospitalization or death in this population	20332801	YS	CAUS
4	Among older adults, consumption of tuna or other broiled or baked fish, but not fried fish, is associated with lower incidence of CHF	15963403	YS	CAUS
5	Increased baked/broiled fish intake may lower HF risk, whereas increased fried fish intake may increase HF risk in postmenopausal women	21610249	YS	CAUS

It is possible that contextual information may affect whether pairs of statements are considered contradictions (e.g. there would be no contradictions between sentences (2) and (4) if sentence (2) only applied to teenagers and fried fish). However, they are considered in isolation and do not take account of their context. This approach ensures that the problem does not become intractable and is common with other work on contradiction detection [6].

### Claim definition and types

The identification of claims, and the contradictions between them, is made more complex by the range of different types of claim that can occur in biomedical literature and various typologies have been proposed. We now define research claim and discuss the types used in the corpus.

*A research claim* can be defined as the summary of the main points presented in a research argument; these points can either introduce new knowledge to readers or update their knowledge on a topic [17]. The claim contains the most important piece of information that authors want to communicate. It represents the research findings or outcomes. In biomedical literature claims tend to summarize the authors findings and occur at the end of the study [17].

Blake [18] identified five types of claims: explicit, implicit, correlations, observations and comparisons. This typology was formulated based on the availability of certain information (facets): two concepts, a change and the basis of the claim. Although the typology provides a framework of how a biomedical claim can automatically be analysed, it was not clear how a judgmental claim such as effectiveness of a drug or a technique can be analyzed, for example, sentence (6) in Table 2.

**Table 2:** Claims typology examples

	Claim	PMID	Type
6	Combined clopidogrel and aspirin overcome single drug resistances, are *safe* for bleeding	22942294	Judgemental
7	Aspirin plus clopidogrel is *more effective* in venous graft patency than aspirin alone in the short term after CABG, but further, long-term study is needed	21050973	Comparative
8	Although a bedtime dose of doxazosin can significantly lower the blood pressure, it can also increase left ventricular diameter, thus increasing the risk of congestive heart failure.	18551024	Excitatory
9	Routine use of postoperative aspirin after coronary artery bypass grafting (CABG) reduces graft failure and cardiovascular events	21146675	Inhibitory
10	In the Spanish Mediterranean area, the presence of antigens B-15 and DQ3 would be associated with advanced DCM	10198739	Neutral

We use another framework [17], which has been constructed from a general perspective rather than specifically for the biomedical domain. The framework consists of four types of claim: factual, recommendation, evaluative and causal. Causal and evaluative claims are the most relevant types for our corpus. Factual claims tend to be generally accepted information and these claims are most commonly found in background sections rather than the conclusion. Recommendation claims often provide recommendations of courses of action supported by the main research claim rather than providing any new information. We describe causal and evaluative claims using examples from the biomedical literature.

*Evaluative claims* occur when an author expresses a judgment about the value of a biomedical concept (e.g. drug, procedure, equipment, gene, protein). This type of claim is often used as an interpretation of evidence presented in the research. It is usually expressed by either making a statement about the properties of a concept (judgment), e.g. sentence (6), or comparing the concept with another, e.g. sentence (7).

*Causal claims* suggest a relationship between two concepts and assert that one concept influences the other. Hashimoto et al. [19] described three types of influences: excitatory, inhibitory and neutral. Excitatory influence indicates a direct activation or enhancement, e.g sentence (8) shows the *doxazosin* had an excitatory influence on *left ventricular diameter*. An inhibitory influence is the opposite of excitatory and indicates direct deactivation or suppression. For example, sentence (9) is a casual claims which asserts that *Routine use of postoperative aspirin* has an inhibitory effect on *graft failure and cardiovascular events*. The final type of causal claim, neutral, is neither excitatory nor inhibitory. For example, sentence (10) asserts a relationship between *presence of antigens B-15 and DQ3* and *advanced DCM (Dilated Cardiomyopathy)* but doesn’t explicitly state whether it is excitatory or inhibitory.

## Corpus construction stages

### Corpus data collection

The corpus was created using research abstracts of studies considered in systematic reviews related to cardiovascular diseases. Cardiovascular diseases have been reported as a major contributor to world mortality and their causes are commonly explored in research papers [20]. Given the volume of research published on the topic we expect to find some contradictory findings.

Four types of cardiovascular disease were chosen: *Cardiomyopathy*, *Coronary artery*, *Hypertensive* and *Heart failure*. The Pubmed search engine was used to retrieve systematic reviews associated with these types. For example, the query “Cardiomyopathy”[title] AND “meta-analysis”[title] was used to search for systematic reviews discussing cardiomyopathy disease, and the same procedure were applied on the other types. The modifier [title] was used to ensure that the search keywords occurred within the title of the article.

**Fig. 1:**
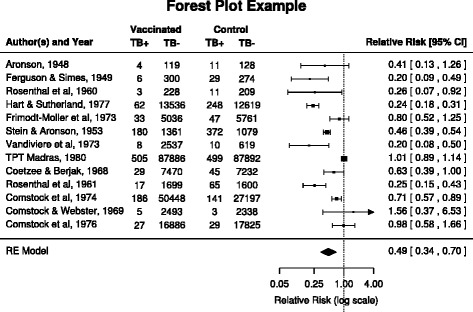
The diagram represents the outcome of studies exploring the effectiveness of a vaccine (BCG) for preventing tuberculosis. Studies that favour the vaccine are shown on the left side of the vertical column while those that favour the placebo are shown on the right side. This diagram shows one study that favoured the placebo (Comstock & Webster, 1969) and two for which no statistically significant difference between the vaccine and placebo could be identified, (TPT Madras, 1980) and (Comstock et al.,1976). The vaccine was favoured in all other studies. The dataset was retrieved from metafor [25], an R package for conducting meta-analyses

Systematic reviews were used since they gather findings from multiple studies related to a defined research question and summarise their results using statistical meta-analysis. Results of the meta-analysis are often presented using a diagram called a forest plot [21] which represents the findings of a set of studies. In each systematic review, the forest plot diagram was examined to determine whether it suggested contradictions between the studies included. If any potential contradictions were identified then all the studies included within the systematic review were included in the corpus. For example, Fig. 1 shows a forest plot in which a single study, (Comstock & Webster, 1969), favours the placebo and consequently this study may contain a claim regarding the effectiveness of the treatment which contradicts those made in the other studies. Although, the difference may not be significant and may not even be reflected in the abstract description, the forest plot diagram is still a good indication that the details discussed in this review may contain contradictory claims.

Table 3 shows a list of abstracts titles; title (11) refers to the systematic review that was collected from MEDLINE; and titles (12-16) are the studies used in that review. These studies were included as candidate datasets since the diagram of the systematic review showed disagreement between at least one of theirs findings.

**Table 3:** A systematic review title and the titles of its associated studies

		Title	PMID
11	Review	Fish consumption and incidence of heart failure a meta-analysis of prospective cohort studies	23489806
12	Study	Incident heart failure is associated with lower whole-grain intake and greater high-fat dairy and egg intake in the Atherosclerosis Risk in Communities (ARIC) study	18954578
13	Study	Intake of very long chain n-3 fatty acids from fish and the incidence of heart failure: the Rotterdam Study	19789394
14	Study	Fatty fish, marine omega-3 fatty acids and incidence of heart failure	20332801
15	Study	Fish intake and risk of incident heart failure	15963403
16	Study	Fish intake and the risk of incident heart failure: the Women’s Health Initiative	21610249

### Question formulation

Biomedical literature contains claims with complex structure, often expressing multiple facts in the same sentence. This may confuse the annotators when annotating contradictory claims in the dataset. To avoid this issue and ensure that annotations correspond with the definition of contradiction being used, a common question shared by claims needs to be identified to ensure that the annotators focus on the same fact when identifying contradictions.

As an attempt to achieve that goal, an annotator with an advanced degree in medicine was asked to use the titles of each systematic review and the studies abstracts included, as information to formulate a suitable question for the group of studies in that review. The annotator was asked to formulate closed questions (i.e. ones that could be answered as either *yes* or *no*) written in simple present tense. He was also asked to ensure that the questions were compiled with the PICO standard [22] to include information about the patient problem or population (P), Intervention (I), comparison (C) and outcomes (O). For example, the question *“In patients with chronic heart disease (P), does bone marrow stem cell transplantation or injection (I), compared to none (C), improve cardiac function (O)?”*.

This approach will enable the annotators to measure the assertion values of claims with respect to the question. Thus, when two claims provides different assertion value or conclusion to a question, they are considered potentially contradictory.

### Corpus annotation

The final stage of corpus construction was to identify and annotate the claims in each abstract. Two annotators were recruited. Each annotator had native-level English fluency, an advanced degree in a field related to medicine and was employed in a medical role (one in an academic department and another in a hospital). Both were familiar with biomedical research literature and evidence-based medical research. The annotators were asked to carry out three tasks: choose a claim, annotate the claim with an assertion value (YS/NO) with respect to the question formulated for the review group, and annotate the claim type (CAUS/EVAL) according the claim types described earlier.

The first task required examining each study abstract and, considering the question that had been formulated from the systematic review, identifying the claim sentence within each abstract that could answer that question. If the abstract contained multiple candidate claim sentences then annotators were asked to select one that corresponds to the overall abstract finding (as represented in the forest diagram) and best describes the contribution of the research relative to the question. After the claim had been identified annotators were asked to mark it as either *YS* (to indicate the claim was an affirmative answer to the question) or *NO* (to indicate that it was not). Finally, the annotators were asked to identify the type of the claim (i.e. causal or evaluative). After each annotation phase the two annotators met to resolve disagreements and decided on the final annotation.

## Results and discussion

Examination of forest plot diagrams lead to the identification of 40 suitable systematic reviews and a question was formulated for each. A total of 397 studies were mentioned in these reviews. These studies were retrieved and annotators asked to identify a claim in each, decide whether this claim agreed with the question that had been formulated and determine the claim type. 19 of the studies were excluded since the annotators were unable to identify a claim that provided a clear answer to the question. No contradictions were identified for 16 of the systematic reviews (i.e. the annotators judged all of the claims in the studies associated with the review as either agreeing or disagreeing with the question). These reviews and the studies associated with them were also excluded.

**Fig. 2:**
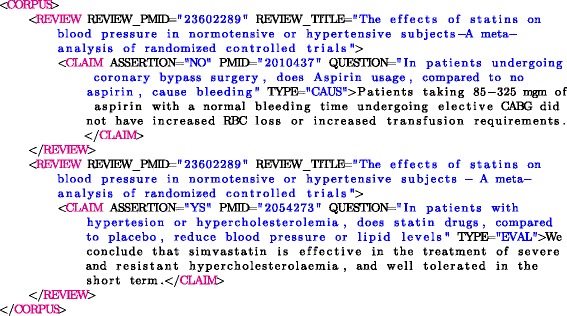
Examples of formatted claims

The final corpus consists of 259 studies used within the 24 systematic reviews that were not excluded. Table 4 shows the number of studies associated with each systematic review and their distribution across the assertion values (*YS* and *NO*). The questions formulated for each systematic review are shown in Table 5. The corpus is formatted in XML as shown in Fig. 2.

**Table 4:** Claims classes and type distribution among the groups

			Assertion		Type	
Topic	Review-PMID	#Abstracts	YS	NO	CAU	EVA
Cardiomyopathy	22498326	4	3	1	2	2
	23623290	9	7	2	3	6
	21556773	15	12	3	12	3
Coronary artery	24035160	5	3	2	2	3
	24135644	20	13	7	13	7
	24036021	4	2	2	4	0
	24212980	20	12	8	14	6
	24039708	18	11	7	17	1
	24172075	7	2	5	0	7
	24090581	8	4	4	2	6
	24040766	5	4	1	5	0
Heart failure	23489806	4	3	1	4	0
	23181122	5	4	1	5	0
	23962886	15	13	2	14	1
	24163234	29	22	7	13	16
	24165432	6	4	2	2	4
	23219304	10	6	4	5	5
	21521728	11	7	4	6	5
Hypertensive	23602289	17	14	3	10	7
	22795718	14	13	1	9	5
	23435582	6	4	2	5	1
	22854636	5	3	2	2	3
	23223091	7	6	1	6	1
	22086840	15	7	8	10	5
TOTAL		259	180	79	165	94

**Table 5:** A list of the 24 questions formulated for the final corpus

Review-PMID	Question
22498326	In patients with HCM, does using imaging technique, compared to conventional techniques, serve as a predictor for adverse prognosis?
23623290	In patients with chronic heart disease, does Bone marrow Stem cell transplantation or injection, compared to none, improve cardiac function?
21556773	In patients with dilated cardiomyopathy, are HLA genes associated with development of Dilated Cardiomyopathy?
24040766	In Han Chinese population, is SNP T-778C of apolipoprotein M associated with risk of developing Diabetes or stroke?
24212980	In patients undergoing coronary bypass surgery, does Aspirin usage, compared to no aspirin, cause bleeding?
24035160	In patients undergoing choronary artery bypass, does the combination of aspirin and clopidogrel, compared to aspirin alone, prevent graft occlusion or improve patency?
24172075	In patients undergoing coronary by pass surgery, is Off-pump, compared to conventional on pump coronary artery bypass grafting, more beneficial?
24135644	In patients with choronary artery disease, is mutation or polymorphisms in endothelial nitric oxide synthase gene associated with CAD or MI or ACS development?
24036021	In patients with atherosclerotic plaque or myocardial infaction, does −463G or −463A polymorphism in MPO gene influence MI or CAD development?
24039708	In patients with coronary artery disease (CAD), is C242T polymorphism of P22(PHOX) gene associated in development of CAD?
24090581	In patients with coronary artery diseases, does combining CABD and CEA, compared with CABG or CEA alone, reduce morbidity?
24165432	In elderly patients with CHF, does physical exercise or cardiac rehabilitation, compared to no exercise, improve cardiac function?
23962886	In patients with heart failure, do statin drugs treatment, compared to non statin drug, treatment improve cardiac function or prevent cardiac morbidity?
23219304	In patients with renal or cardiovascular disease, does treatment with ACE inhibitors, compared with placebo, improve renal function or protect against cardiovascular incidents respectively?
23181122	In the elderies, is n-3 fatty acid from fish intake associated with reduction in risk of developing heart failure?
23489806	In the elderlies, does omega 3 acid from fatty fish intake, compared with no consumption, reduce the risk of developing heart failure?
24163234	In patients with CHF, does care giving or teleguidiance-telecare, compared to usual care, reduce morbidity?
21521728	In patients with advanced diabetes, does treatment with antihypertensives, compared with placebo, improve renal function or protect againct cardiovascular incidents?
22854636	In patients with hypertension, does revascularisation, compared with medical therapy, improve blood pressure?
22795718	In patients with hypertension, does treatment with ACE inhibitors, compared to placebo, reduce risk of cardiovascular event or improve blood pressure?
23602289	In patients with hypertesion or hypercholesterolemia, does statin drugs, compared to placebo, reduce blood pressure or lipid levels?
23435582	In women with pre-eclampsia, does treatment with L Arginine, compared to placebo, reduce blood pressure or pre-eclampsia?
22086840	In women with pre-eclampsia, is Polymorphism in angiotensin gene associated with pre-eclampsia?
23223091	In women with pre-eclampsia, is mutation in renin-angiotensin gene associated with pre-eclampsia?

The annotators were asked to complete three tasks: identify a single claim within each abstract, determine whether that claim agreed with the research question or not, and annotate the claim type (CAUS/EVAL).

Inter-annotation agreement for the claim identification task was 92 %. The main reason for disagreement was cases where there were multiple claims in the same study abstract that potentially answer the question formulated for the systematic review. For example, Table 6 shows two sentences, (17) and (18), extracted from an abstract that potentially answer the question *“In women with pre-eclampsia, is polymorphism in angiotensin gene associated with pre-eclampsia?”*. In such cases the annotators were asked to prefer sentences in the conclusion sections of the abstracts.

**Table 6:** Potential answers to a formulated question from the same abstract

	Sentence	PMID	Value	Type
17	The frequency of T allele of angiotensinogen T174M gene was slightly increased, but not significantly, in preeclampsia (0.11) than in controls (0.07)	15082899	YS	CAUS
18	In conclusion, a molecular variant of ACE, but not angiotensinogen, gene is associated with preeclampsia in Korean women	15082899	YS	CAUS

Agreement for the second task, determining whether the claim agreed with the question or not, was very high (97 %). The disagreements that did occur arose from claims that did not provide a conclusive answer to the relevant question. This problem was generally avoided by formulating a question for each systematic review but in some cases multiple inferences can be derived from the same claim. For example, the question *“In the elderly, is n-3 fatty acid from fish intake associated with reduction in risk of developing heart failure?”* asked about the association of *n-3 fatty acid from fish* and the risk of *developing heart failure* but did not specify the type of fish. Table 7 shows multiple inferences derived from claims (4) and (5) in Table 1 (inferences (4a) and (4b) from claim (4) and inferences (5a) and (5b) from claim (5)). These inferences differ in their agreement with the question. In such situations annotators were asked to choose the inference that is the best fit for the question. In this case, inference (4a) was used for claim (4) since it is more general than the alternative (4b). Similarly, inference (5a) was used for claim (5) rather than (5b) since the second referred to a restricted population (postmenopausal women).

**Table 7:** Multiple inferences derived from two claims

	Inference	PMID	Value
4a	consumption of tuna or other broiled or baked fish is associated with lower incidence of CHF	15963403	YS
4b	fried fish is not associated with lower incidence of CHF	15963403	NO
5a	Increased baked/broiled fish intake may lower HF risk	21610249	YS
5b	Increased fried fish intake may increase HF risk in postmenopausal women	21610249	NO

Lower agreement (86 %) was obtained for the final task, annotation of claim type. The main cause of disagreement were claims that could potentially be simultaneously interpreted as causal or evaluative. For example, the claim *“These results suggest that HLA-DR4 antigen may be a genetic marker for susceptibility to dilated cardiomyopathy”* can be considered a causal claim since it describes an association relation between the two concepts *HLA-DR4 antigen* and *dilated cardiomyopathy*. But it can also be evaluative since the author is evaluating the effectiveness of that gene as a genetic marker. Annotators were reminded that evaluative claims should express a judgement, which is not the case here and it was consequently annotated as a causal claim.

The high inter-annotator agreement figures indicate that the annotation tasks are well-defined and that the annotations are reliable and form a sound basis for future studies. Although agreement for the claim type identification task is lower than the others, the information may still be useful for further exploration.

Automatic identification of contradictory claims is a difficult problem and a number of challenges were identified during the construction of our corpus. Claims tend to appear at the end of abstracts and consequently authors often use shorter forms such as acronyms, for example *“Our observations indicate a significant relationship between p22phox C242T and PARP-1 Val762Ala polymorphisms, CAD and its severity, but not with occurrence of MI in T2DM individuals with significant coronary stenoses”*. This complicates the process of identifying claims, particularly since acronyms are often ambiguous in biomedical text [23, 24].

Identifying connections between statements is also complicated by authors’ use of alternative terms. For example, *statin*, *atorvastatin* and *rosuvastatin* were all used to refer to drugs that lowers cholesterol levels in studies included in the corpus.

The corpus developed in this research could be used as a resource for researchers to explore the problems of identifying, analysing and resolving contradictory claims made in the biomedical literature. For example, it could be used to build a machine learning system that discriminates between claims that agree or disagree with a query, where contradiction occurs between them when they provide different answers to the same query. Moreover, the construction methodology described in this paper could be applied to construct other corpora containing potentially contradictory claims.

## Conclusions

The contradictory claims found in biomedical literature present a challenge to evidence-based evaluation into the effectiveness of approaches. Automatic identification, analysis and resolution of these claims would be useful for those that rely on this literature.

This paper described the development of a corpus containing contradictory claims found within Medline. Systematic reviews were used to identify studies that contain contradictory statements regarding particular research questions. Claims within the studies were identified and annotated. Analysis shows that the agreement between annotators is reliable, suggesting that the information in the corpus will be useful for those who wish to explore this problem. The corpus construction methodology could be applied to other topics in the biomedical domain.

The corpus can be accessed via: http://staffwww.dcs.shef.ac.uk/people/M.Stevenson/resources/bio_contradictions/.
